# Very Young Infants’ Sensitivity to Consonant Mispronunciations in Word Recognition

**Published:** 2023

**Authors:** Caroline Beech, Daniel Swingley

**Affiliations:** Department of Psychology, 425 S. University Ave., Philadelphia, PA 19104 USA; Department of Psychology, 425 S. University Ave., Philadelphia, PA 19104 USA

**Keywords:** language acquisition, word recognition, phonological representations, mispronunciation sensitivity

## Abstract

Before they start to talk, infants learn the form and meaning of many common words. In the present work, we investigated the nature of this word knowledge, testing the specificity of very young infants’ (6–14 months) phonological representations in an internet-based language-guided-looking task using correct pronunciations and initial-consonant mispronunciations of common words. Across the current sample (n=78 out of 96 pre-registered), infants’ proportion looking to the target (named image) versus the distracter was significantly lower when the target word was mispronounced, indicating sensitivity to phonological deviation. Performance patterns varied by age group. The youngest group (6–8 months, n=30) was at chance in both conditions, the middle group (9–11 months, n=21) showed significant recognition of correct pronunciations and a marginal mispronunciation effect, and the oldest age group (12–14 months, n=27) demonstrated the mature pattern: significant recognition and a significant mispronunciation effect. Ongoing work is completing the pre-registered sample size.

## Introduction

Mature language comprehension relies on accurate representations of words’ phonological forms. For example, adult English speakers know that the word “dog” starts with /d/ and that a /t/ is unlikely to signal a speaker starting to say “dog.” But when infants start learning the words of their language, they lack the mature phonological categories, they might not know that phonological distinctions signal lexical distinctions, and their memory for individual words might be vague. So how should we think about the earliest words?

One argument holds that children encode only a perfunctory phonetic representation of words, because with their tiny vocabularies, there is little functional need for them to retain the phonetic details of words. If the only words you know are, say, “banana” and “shoe,” you could tell them apart even if you only knew that one started with /b/ and had three syllables, and were only able to represent additional distinctions as the lexicon demanded it (e.g., Charles-Luce & Luce, 1995; but see Cui, 2020). Infants who don’t speak yet also lack the articulatory experience of trying to produce words interpretably, which would otherwise be another impetus for accurate phonetic representation (e.g, Vihman & Croft, 2007). Furthermore, word learning begins while infants are still refining their knowledge of the phonological categories of their language (e.g., Bergelson & Swingley, 2012; Werker & Tees, 1984). Thus, an initial period of phonologically imprecise representations might be expected.

On the other hand, existing research examining the phonological representations of infants over about 12 months suggests that they are already sensitive to one-phoneme mispronunciations, with no increase in sensitivity over the second year of life (Von Holzen & Bergmann, 2021). In the present work, we investigate the nature of infants’ phonological representations in the first year of life through to early in the second, testing infants from three different age groups: 6- to 8-month-olds, 9- to 11-month-olds, and 12- to 14-month-olds.

### Infants’ Word Form Representations

From birth, infants appear to be capable of perceptually discriminating speech sounds from many different phonological categories. For instance, 1- and 2-month-old infants are already sensitive to the differences in voice onset time and formant transitions that distinguish the phonemes /b/ and /p/ or /b/ and /w/ in English (Eimas et al., 1971; Eimas & Miller, 1980). Adding to this picture of precocious speech sound perception, slightly older infants prefer listening to lists of word forms that are familiar from home or laboratory exposure over similar sounding one-phoneme substitutions, such as “labbit” instead of “rabbit” (e.g., Hallé & de Boysson-Bardies, 1996; Jusczyk & Aslin, 1995). However, neither of these effects on the perception side reveal what kind of phonological representations infants use in word comprehension. What phonological or acoustic information do infants store when they acquire words as lexical items with associated meanings?

To probe what infants know about the phonological forms of words early in the process of language learning, Swingley and Aslin (2000, 2002) presented English-learning infants from 14 to 23 months with correct pronunciations (e.g., “dog”) and deliberate mispronunciations (e.g., “tog”) of familiar words in a language-guided-looking task (Fernald et al., 1998). On each trial, participants saw a pair of images (e.g., a baby and a dog) and heard a sentence naming one of the images (e.g., “Where’s the *dog*?” or “Where’s the *tog*?”). To index mispronunciation sensitivity, and thus the phonological specificity of infants’ lexical representations, Swingley and Aslin compared infants’ looking to the target image in the correct pronunciation trials to target looking in the mispronunciation trials. They found that although infants looked longer at the target than at the distracter in both types of trials, correct pronunciations elicited significantly more target looking than one-phoneme mispronunciations, suggesting that these children’s representations of familiar words were already phonologically well specified. The same paradigm has since been employed to investigate the specificity of infants’ word knowledge across a variety of phonological features, ages, and languages (e.g., Altvater-Mackensen et al., 2014; Mani & Plunkett, 2010; Ramon-Casas et al., 2009; White & Morgan, 2008).

A recent meta-analysis (Von Holzen & Bergmann, 2021) combined the data from the past twenty years of mispronunciation experiments to investigate, among other questions, whether mispronunciation sensitivity changes with age. Von Holzen and Bergmann found robust evidence that infants are sensitive to mispronunciations, and that across studies, this ability was not modulated by age. This might suggest that infants have mature, well-specified phonological representations of familiar words even in the earliest stages of word recognition. However, little research has explored mispronunciation sensitivity in very young infants. Substantial recent evidence shows that infants as young as 9 or 10 months (Nomikou et al., 2019; Parise & Csibra, 2012; Syrnyk & Meints, 2017) or even 6 or 7 months (Bergelson & Swingley, 2012, 2015) understand several common words. However, only 1 out of the 32 papers included in the meta-analysis (Bergelson & Swingley, 2018) tested infants under 12 months.

Bergelson and Swingley (2018) used a between-subjects design comparing recognition of correct pronunciations and vowel mispronunciations (e.g., “banana” versus “banoona”) in infants from 6 to 14 months of age. Between-subjects comparison is difficult at this age because performance is so variable, but overall Bergelson and Swingley’s results suggest that the vowels in early-learned words may not be precisely represented before 11 months, with the youngest infants exhibiting equally high recognition (increase in target looking) in both groups (correct pronunciations versus mispronunciations). Thus, this study offers some support for the idea that infants’ earliest word recognition involves imprecise phonological representations. Here we build on this work, investigating very young infants’ sensitivity to consonant mispronunciations using a within-subjects design.

### Present Work

In this work, we sought to determine at what age infants come to accurately represent the consonants within words. We tested infants from three different age groups, 6 to 8 months, 9 to 11 months, and 12 to 14 months, in a language-guided-looking procedure using mispronunciations of familiar words. If, at a particular age, infants recognize words (i.e., look more to the target image when it is labeled) but are insensitive to consonant mispronunciations, this would suggest that language learning involves an early period of imprecise, holistic representations of how words sound, before words’ consonants, which are thought to anchor lexical processing (e.g., Nazzi & Cutler, 2019), come to be represented. Thus, the results of this work have important implications for theories of language learning. Whatever computations language learning involves must be compatible with the phonological representations that infants have available.

## Method

### Participants

Participants in the current sample were 30 6- to 8-month-olds, 21 9- to 11-month-olds, and 27 12- to 14-month-olds. Ongoing work adds infants to these age groups to achieve our pre-registered sample size of 32 infants per age group. All infants were carried full-term, raised in a monolingual English environment (at least 75% English exposure, based on parental estimate), and had no reported hearing problems. An additional 61 infants were tested but excluded from the final sample because of equipment failures (n=10), parental interference with the task (n=1), or because they failed to contribute at least 8 trials to each condition (n=50)^1^. Following our pre-registration, trials were excluded when infants failed to fixate the images for at least 2/3 of a second in the analysis window, when they belonged to a sequence of two or more consecutive trials where the infant only ever fixated one side of the screen, or when technological issues or background noise made it impossible to confirm the timing of stimulus presentation.

### Materials

We tested correct pronunciations and mispronunciations of 16 target words (Table 1) that are frequent in speech to children and easily depicted. Mispronunciations were created by changing each word’s initial consonant (or consonant cluster, in the case of “spoon”). As in previous studies using this method, on each trial, participants heard a sentence highlighting one of the target words (e.g., “Look at the ball!”) while viewing two pictures, one of which matched the spoken sentence. Items were paired such that the same two images (e.g., the ball image and the shoe image) always appeared together.

To create the auditory stimuli, we recorded a female native speaker of American English who produced each sentence in clear, child-directed speech, using the same prosody for the correct pronunciations and the mispronunciations. We recorded multiple instances of each sentence and selected the stimuli so that correct pronunciations and their corresponding mispronunciations were as closely matched as possible, qualitatively and in duration (M = 0.70 s, SD = 0.10 vs. M = 0.69 s, SD = 0.09). During the experiment, each sentence was preceded by a bell sound signaling the start of trial followed by two seconds of silence. This bell sound, in combination with two metronome clicks embedded in the silent period, allowed us to confirm the timing of stimulus presentation when reviewing participants’ recordings.

The visual stimuli were photographs of objects against a gray background, presented side by side on participating families’ computers. Each image measured approximately 1/16 of the screen. Children were seated at eye-level with the pictures, on their parent’s lap or in a high-chair or other supported position. Stimulus presentation across trials was pseudo-randomized such that the target pictures appeared on the left and the right equally often, but never more than twice in a row on the same side, and not covarying with condition (correct pronunciation versus mispronunciation) or carrier phrase (“Look at the” versus “Find the”).

### Procedure

All participants were tested at home over the internet using the PCIbex online experiment platform (Zehr & Schwarz, 2018). After video-calling with our lab manager and filling out the study consent form, parents were sent a link to complete the experiment at a time convenient for them and their child. Thus, parents’ personal computers were used for experimental presentation and participant recording. Parents were told to seat their child in front of the computer screen so that the child’s face would be clearly visible and centered in the webcam recording, and asked to refrain from speaking, pointing, or otherwise directing the child during the experiment. Following the experiment, parents also completed the MacArthur-Bates CDI (Fenson et al., 1994) and an optional demographic questionnaire.

**Figure 1:**
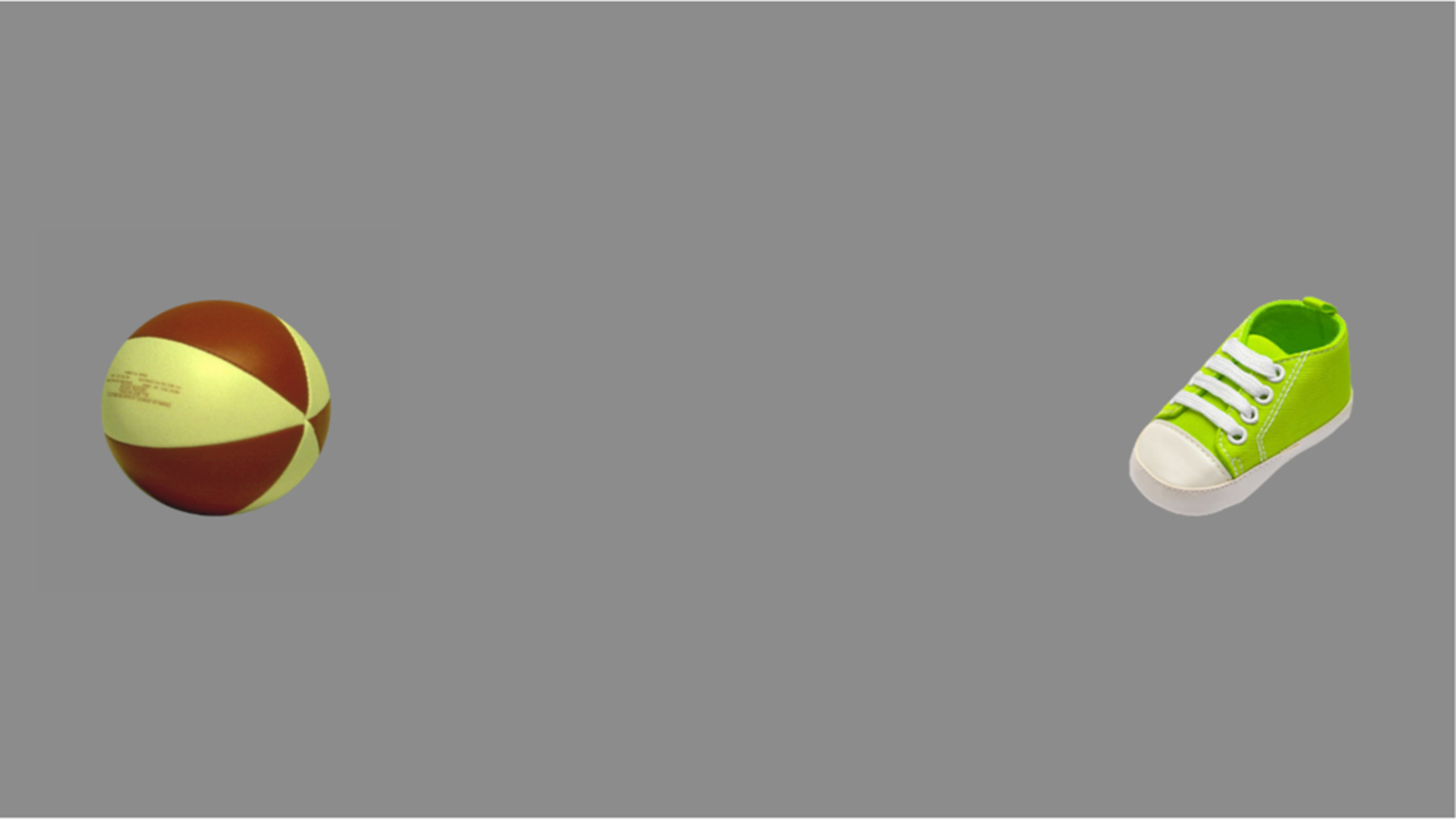
Example trial. Accompanying this visual display, infants would hear a sentence naming one of the pictures using either a correct pronunciation (e.g., “Find the ball!”) or initial-consonant mispronunciation (e.g., “Find the chall!”).

At the beginning of the experiment, participants saw five calibration trials, in which a flower-shaped color wheel appeared suddenly on the left or right side of the screen, and four warm-up trials featuring words and images not used in the rest of the experiment (e.g., a fish image and a sock image accompanied by the sentence “Where’s the sock?”), which served to acclimate infants to the task. These were followed by 32 experimental trials (see Figure 1 for an example). Each of the 16 target words (Table 1) was presented once as a correct pronunciation (e.g., “ball”) and once as a mispronunciation (e.g., “chall”). Half the words appeared first in their correctly pronounced form and half in their mispronounced form, with trials featuring the same pair of images spread out throughout the experiment.

### Data Preparation

In order to measure looking to the target and distracter, participant recordings were manually coded offline by the first author and trained research assistants. We examined the recordings frame by frame (1 frame = 33 ms) using ELAN (2022) and coded for each frame whether the infant was looking toward the left side of screen, the right side of screen, away from the screen, or in transition. Left and Right codes were then converted to Target and Distracter codes based on target side. Following previous work (e.g., Bergelson & Swingley, 2012) and our pre-registration, we analyzed target and distracter looking from 367 ms to 3500 ms after target-word onset.

## Results

Here we report preliminary results from our sample thus far (see Participants) on 1) how well infants recognized words when they were pronounced correctly, and 2) how infants’ target looking was affected by condition (correct pronunciations versus mispronunciations). If even very young infants have precise phonological representations of words, we would expect to see successful recognition of correct pronunciations, and reduced target looking in the mispronunciation trials. If infants have less precise, more holistic representations of words, however, we would expect them to accept loose matches (e.g., “nottle” for “bottle”) with no significant decrement in target looking.

### Recognition

We assessed infants’ recognition of item pairs using mixed effects logistic regression. In this analysis, the underlying idea is that if infants know a word (e.g., “ball”), then they should look more to the image when it is named (e.g., “Look at the ball!”) than when the other image in the pair is named (e.g., “Look at the shoe!”). Previous work (e.g., Bergelson & Swingley, 2012) has assessed word recognition in a similar manner using t-tests on the subject means (for each subject, for the average item pair, what was the proportion looking to X when X was the target, minus when X was the distracter). The advantage of using a mixed effects logistic regression model, however, is that it uses the trial-level proportion looking data and accounts for repeated measurements from subjects and from items at the same time. On average across the sample, the regression model revealed a significant effect of x_named, a binary variable indicating whether the image was the target or the distracter on that trial (β = 0.25, SE = 0.06, *p* < 0.001)^2^.

**Figure 2:**
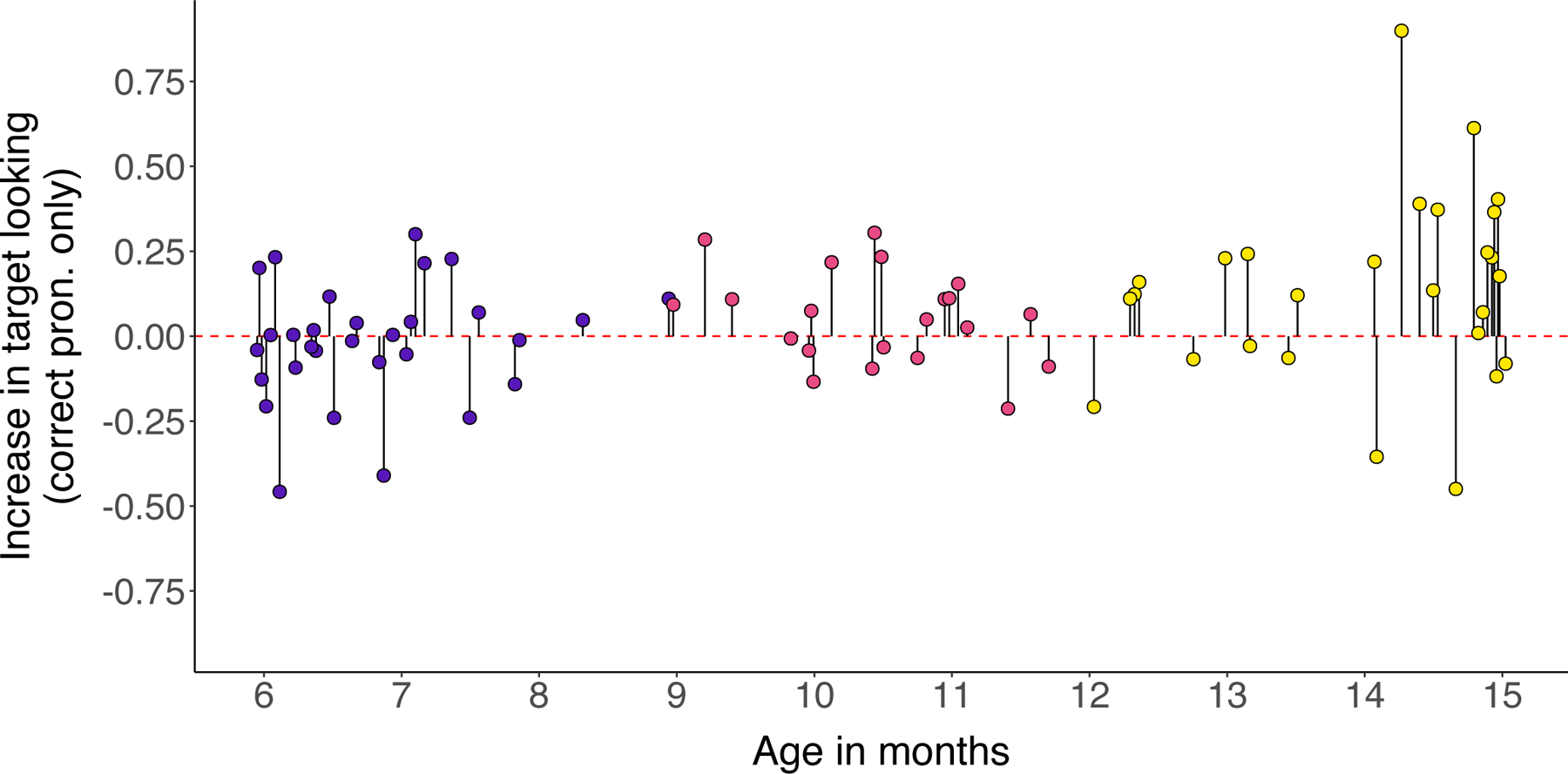
Increase in target looking by age for correct pronunciation stimuli only. The y-axis indicates infants’ mean difference scores in the 367-to-3500-ms window across items. As age increases, there are more points above zero, or infants who looked more to an image when it was named than when the other image in the pair was named, demonstrating successful recognition of the correct pronunciations.

Using the same mixed effects logistic regression model, we also found a significant interaction (β = 0.04, SE = 0.02, *p* = 0.020) between x_named and continuous age, with older infants showing better word recognition (Figure 2). We then tested for evidence of recognition in each of our three age groups, using pairwise comparisons of the estimated marginal means, substituting age group for continuous age in the model. In this analysis, the 6- to 8-month-olds showed no significant recognition effect (β = 0.23, SE = 0.18, *p* = 0.205), but both the 9- to 11-month-olds (β = 0.51, SE = 0.21, *p* = 0.016) and the 12- to 14-month-olds (β = 0.76, SE = 0.18, *p* < 0.001) showed significant evidence of recognition. More traditional t-test analyses, which were also included in our pre-registration, were consistent with all of these results.

### Mispronunciation Sensitivity

**Figure 3:**
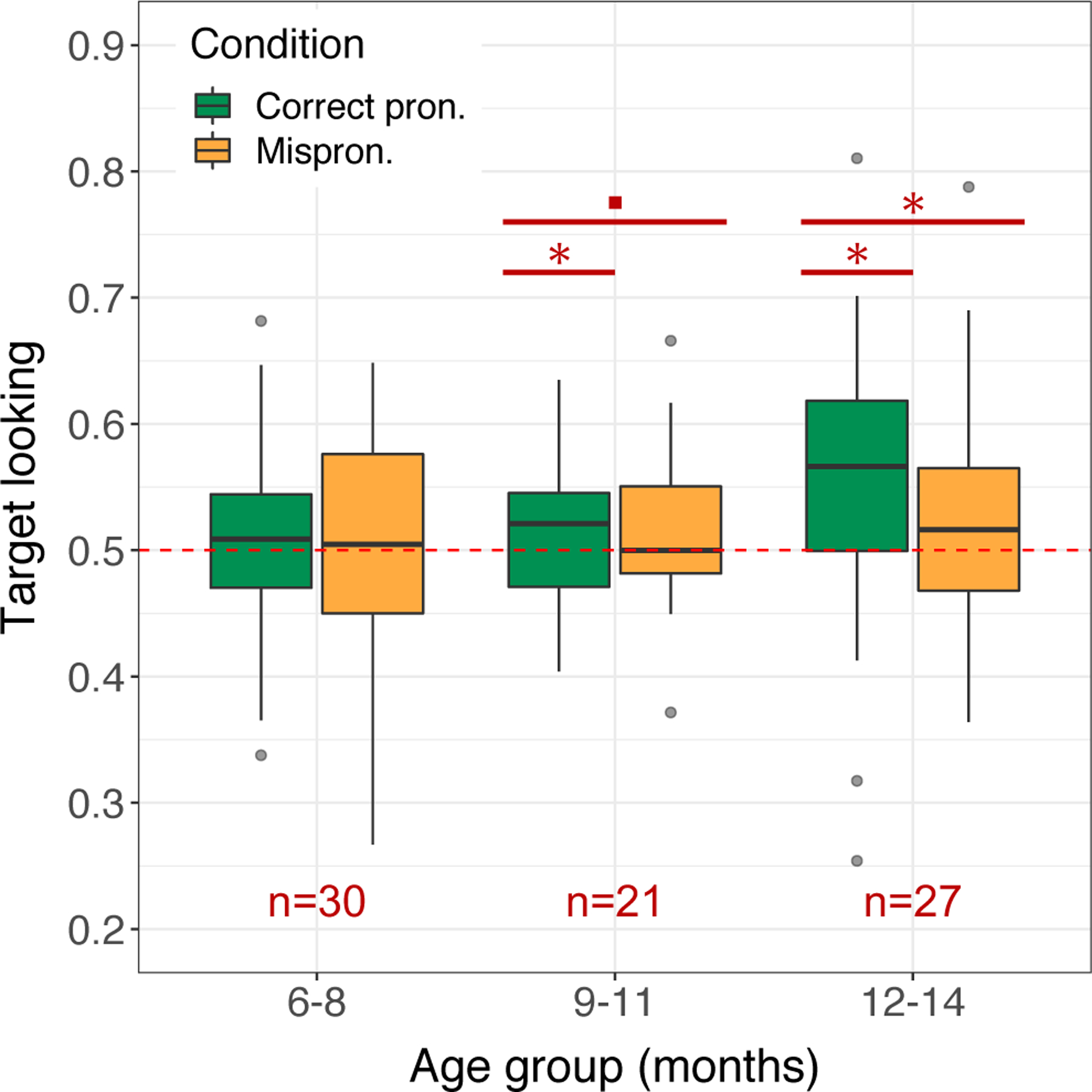
Target looking elicited by correct pronunciation stimuli versus mispronunciation stimuli for each age group. On average across the sample, target looking was significantly lower in response to mispronunciations. Significance annotations indicate ages at which there was a significant (*, p<0.05) or marginal (., p<0.1) effect of condition, and age-condition combinations where target looking was significantly above chance according to a mixed effects logistic regression model.

To measure infants’ sensitivity to mispronunciations and thus the phonological specificity of their lexical representations, we compared infants’ proportion target looking in the correct pronunciation and mispronunciation trials (Figure 3) using mixed effects logistic regression. The full model formula was target_looking ~ condition*age_group + loc_at_onset_is_target + salience + target_side + condition:loc_at_onset_is_target + (condition + 1 | subject) + (condition + 1 | item), using the same naming and contrast coding conventions as before. This model allowed us to assess mispronunciation sensitivity on average across the sample, and in each of our three age groups.

**Figure 4:**
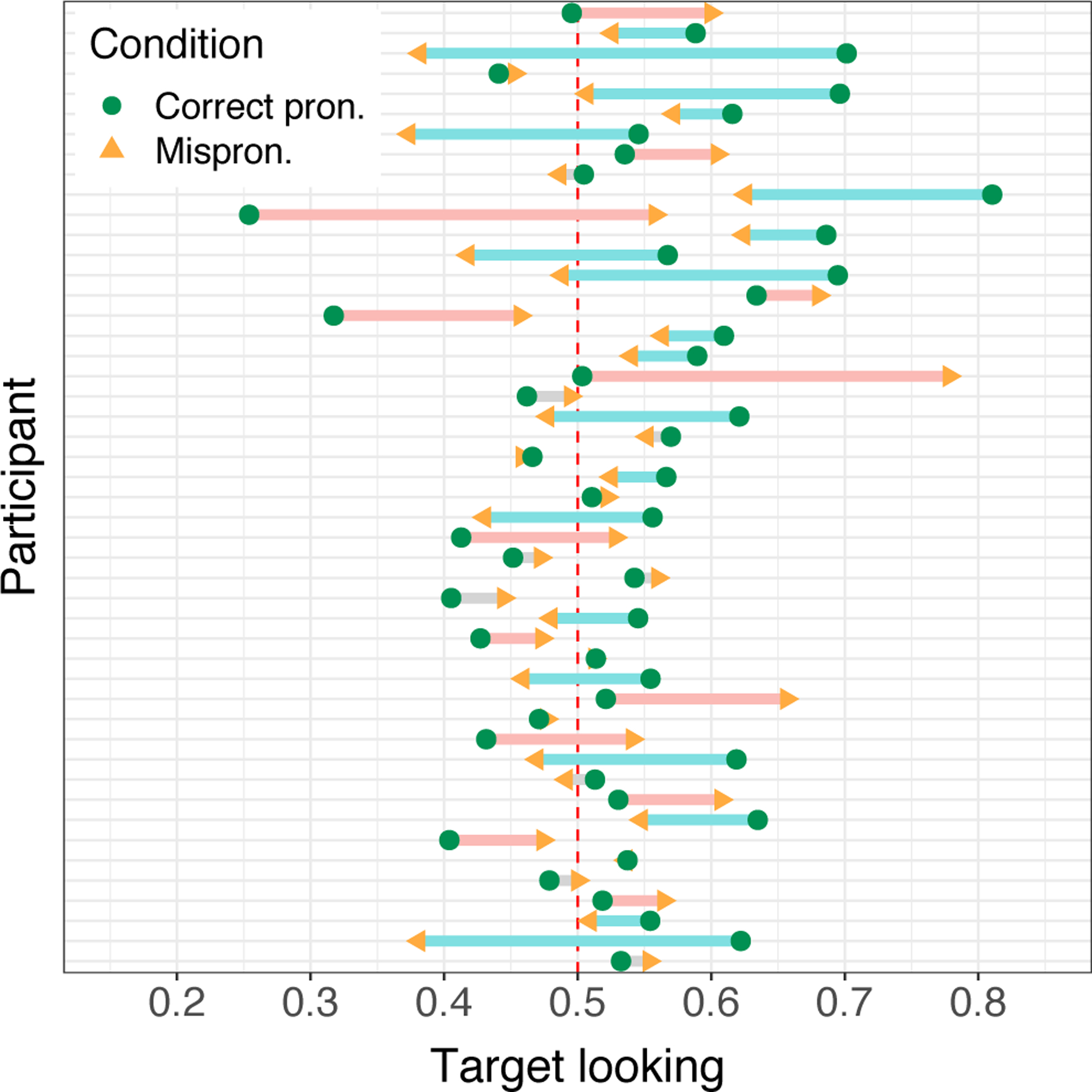
Mispronunciation effect by participant (9–14 months only, ordered by age). Each participant is represented by an arrow pointing from their correct-pronunciations target looking (green circle) to their mispronunciations target looking (orange arrowhead). Arrows are colored by whether the mispronunciation effect was negative (blue), positive (red), or close to 0 (gray).

On average across the sample, there was a significant effect of condition on proportion target looking (β = 0.16, SE = 0.05, *p* = 0.002), with mispronunciation stimuli eliciting less target looking than correct pronunciation stimuli. However, there was considerable variability by age group (Figure 3) and by participant (Figure 4). In the 6- to 8-month-old group, there was no significant mispronunciation effect (β = 0.20, SE = 0.17, *p* = 0.235). However, this result reveals little about these infants’ phonological representations of words because proportion target looking was not significantly above 50% in either condition (correct pronunciations: P̂ = 0.55, 95% CI = [0.49, 0.61]; mispronunciations: P̂ = 0.50, 95% CI = [0.44, 0.56]), and we found no significant evidence of recognition in this age group (see Recognition). In the 9- to 11-month-old group, by contrast, proportion target looking was significantly above 50% in the correct pronunciation trials (P̂ = 0.59, 95% CI = [0.52, 0.65]), and there was a marginal effect of correct pronunciations versus mispronunciations (β = 0.34, SE = 0.20, *p* = 0.080). Finally, the 12- to 14-month-olds exhibited correct pronunciations target looking significantly above 50% (P̂ = 0.62, 95% CI = [0.56, 0.67]), and a significant mispronunciation effect (β = 0.41, SE = 0.16, *p* = 0.011).

## Discussion

The present work represents one of the first investigations of the phonological representations that very young infants bring to bear during word comprehension. Following previous work, we measured infants’ sensitivity to deliberate mispronunciations in a language-guided-looking task. In our analysis of the current sample, we found that infants 12 to 14 months of age, and across the sample on average, demonstrated a significant mispronunciation effect, looking less to the target image when the initial consonant in the target word was mispronounced. Ongoing work adds infants to the 9- to 11-month-old age group (current n=21, out of 32 pre-registered). Given our current recruiting and testing rates, we expect to complete the sample by mid-June, providing a conclusive answer as to whether this younger age group is equally sensitive to small mispronunciations.

The mispronunciations in this study were all mispronunciations of the initial consonant, but they were not designed to systematically test specific feature changes (voicing, manner of articulation, and place of articulation). Indeed they all involved changes to multiple features of the initial consonant. Work with older children (e.g., Altvater-Mackensen et al., 2014; White & Morgan, 2008) has explored how changing single features of consonants affects word recognition, but for this first investigation of children under 12 months, we chose to test the kind of consonant mispronunciations we thought would have the largest effect on recognition, if these infants are representing words’ phonological forms with any precision. If the results from the full sample show that young infants are sensitive to these coarser mispronunciations, future studies with infants of this age could use more fine-grained mispronunciations to examine which details in particular are represented.

While not the focus of the investigation, this work also provides further evidence that young infants’ recognition of words is robust across speakers. Infants in this study all heard the same materials pre-recorded by a female research assistant, rather than produced live by their parent (e.g., Bergelson & Swingley, 2012). Nevertheless, infants in both the 9- to 11-month-old and 12- to 14-month-old age groups showed significant recognition of the correct pronunciation stimuli, successfully generalizing to an unfamiliar talker’s speech.

Unexpectedly, the 6- to 8-month-old group we tested failed to demonstrate significant recognition of the correct pronunciations. Proportion target looking in this condition-age combination was not significantly above chance (50%), and which image was named had no significant effect on infants’ proportion image looking. Performance at this age is variable (e.g., Bergelson & Swingley, 2018), thus we might have failed to find an effect by chance. However, another possibility is that these 6- to 8-month-olds performed less well due to our testing method: testing infants at home on parents’ personal computers over the internet, as opposed to traditional in-lab testing. If so, then our results could have important practical implications for future online versus in-lab research with infants at these ages.

In summary, this work provides new data about very young infants’ word recognition and phonological representations. While several recent studies have demonstrated that infants recognize words far earlier in development that previously thought (Bergelson & Swingley, 2012, 2015; Nomikou et al., 2019; Parise & Csibra, 2012; Syrnyk & Meints, 2017), little research has yet been done on infants’ phonological representations at these early ages. Thus, this work will help to guide theories of language learning, offering new insight into the specificity of infants’ phonological representations in a referential context.

## Figures and Tables

**Table 1:** Target word stimuli. MPs varied from CPs only in the initial consonant(s).

Correct pron. (CP)	Mispron. (MP)	Paired Word
baby	raby	dog
ball	chall	shoe
book	sook	diaper
bottle	nottle	kitty
car	lar	phone
kitty	yitty	bottle
cup	wup	shirt
diaper	myper	book
dog	mog	baby
foot	yoot	window
leg	feg	spoon
phone	roan	car
shirt	lirt	cup
shoe	woo	ball
spoon	croon	leg
window	kindow	foot

## Footnotes

1 This higher than usual dropout rate is likely a consequence of testing infants in the home over the internet. While this testing method made it possible for families to participate during the COVID-19 pandemic, families’ home environments and personal computers sometimes introduce more distractions and equipment failures than in-lab testing.

2 The full model formula was x_looking ~ x_named*age + loc_at_onset_is_x + salience + x_side + x_named:loc_at_onset_is_x + (x_named + 1 | subject) + (x_named + 1 | item). The three control predictors refer to (respectively) whether the infant was looking at the image at target word onset, how much the infant preferred the image in the period before target-word onset across all trials using that image pair, and whether the image was on the right or the left. x_named and loc_at_onset_is_x were deviation coded, and age was centered (continuous and measured in days but transformed into months by dividing by 30.42).
